# Adalimumab for the treatment of fistulas in patients with Crohn’s disease

**DOI:** 10.1136/gut.2008.159251

**Published:** 2009-02-05

**Authors:** J-F Colombel, D A Schwartz, W J Sandborn, M A Kamm, G D’Haens, P Rutgeerts, R Enns, R Panaccione, S Schreiber, J Li, J D Kent, K G Lomax, P F Pollack

**Affiliations:** 1 Hôpital Claude Huriez, Centre Hospitalier Universitaire de Lille, Lille, France; 2 Gastroenterology, Vanderbilt University Medical Center, Nashville, Tennessee, USA; 3 Mayo Clinic, Rochester, Minnesota, USA; 4 St Vincent’s Hospital and University of Melbourne, Melbourne, Australia; 5 Imelda Ziekenhuis, Bonheiden, Belgium; 6 University Hospital of Gathuisberg, Leuven, Belgium; 7 St Paul’s Hospital, University of British Columbia, Vancouver, British Columbia, Canada; 8 University of Calgary, Calgary, Alberta, Canada; 9 Christian-Albrechts University, Kiel, Germany; 10 Abbott Laboratories, Parsippany, New Jersey, USA; 11 Abbott Laboratories, Abbott Park, Illinois, USA

## Abstract

**Objective:**

To evaluate the efficacy of adalimumab in the healing of draining fistulas in patients with active Crohn’s disease (CD).

**Design:**

A phase III, multicentre, randomised, double-blind, placebo controlled study with an open-label extension was conducted in 92 sites.

**Patients:**

A subgroup of adults with moderate to severely active CD (CD activity index 220–450) for ⩾4 months who had draining fistulas at baseline.

**Interventions:**

All patients received initial open-label adalimumab induction therapy (80 mg/40 mg at weeks 0/2). At week 4, all patients were randomly assigned to receive double-blind placebo or adalimumab 40 mg every other week or weekly to week 56 (irrespective of fistula status). Patients completing week 56 of therapy were then eligible to enroll in an open-label extension.

**Main Outcome Measures:**

Complete fistula healing/closure (assessed at every visit) was defined as no drainage, either spontaneous or with gentle compression.

**Results:**

Of 854 patients enrolled, 117 had draining fistulas at both screening and baseline (70 randomly assigned to adalimumab and 47 to placebo). The mean number of draining fistulas per day was significantly decreased in adalimumab-treated patients compared with placebo-treated patients during the double-blind treatment period. Of all patients with healed fistulas at week 56 (both adalimumab and placebo groups), 90% (28/31) maintained healing following 1 year of open-label adalimumab therapy (observed analysis).

**Conclusions:**

In patients with active CD, adalimumab therapy was more effective than placebo for inducing fistula healing. Complete fistula healing was sustained for up to 2 years by most patients in an open-label extension trial.

ClinicalTrials.gov Identifier: NCT00077779 and NCT00195715.

Fistulising disease complicates Crohn’s disease (CD) in up to 40% of patients.^1^^–^3 Fistulas rarely heal spontaneously and usually require medical therapy or surgery.^1^ Antibiotics and immunosuppressive agents have been widely used for treatment, although their efficacy for the sustained closure of fistulas has not been proved.^1^ Tumour necrosis factor (TNF) antagonists specifically target the elevated concentrations of TNF that contribute to the pathological inflammation in CD and represent a significant therapeutic advance in the treatment of patients with CD. Infliximab, a chimeric monoclonal antibody to TNF, has been shown to be effective for the treatment of fistulas.^4^ In patients with an initial response to infliximab induction therapy, there is an increased likelihood of sustained response if infusions are continued every 8 weeks.^5^ Clinical trials have demonstrated that adalimumab, a self-injected fully human monoclonal antibody to TNF, is effective for the induction and maintenance of remission in patients with moderate to severe CD.^6^^–^10 In addition, in patients with CD who have lost response to or have become intolerant of infliximab, adalimumab has been demonstrated to be safe and efficacious in regaining a medical response.^11^

The Crohn’s Trial of the Fully Human Antibody Adalimumab for Remission Maintenance (CHARM)^6^ was a large, phase III, randomised, double-blind, placebo controlled, 56-week study of patients with moderate to severe CD who may or may not have received TNF antagonist therapy previously. The primary objective was to assess the benefit of two adalimumab dosing regimens in maintaining clinical remission at 26 and 56 weeks. Among patients who responded to adalimumab, both dosing regimens (40 mg of adalimumab every other week (eow) and weekly) were statistically significantly more effective than placebo in maintaining remission to 56 weeks. Overall efficacy in fistula closure was also assessed, with significant effects of adalimumab therapy on fistula closure observed at both weeks 26 and 56.^6^ The objectives of the present analysis of patients with fistulas in CHARM were as follows: (1) to describe fully the demographics, disease characteristics and safety outcome of these patients with fistulas; (2) to describe a new statistical approach that was developed to provide a more accurate and longitudinal approximation of fistula burden than was possible with previous approaches by calculating the number of draining fistulas per day for each individual patient and (3) to evaluate the 2-year maintenance of fistula healing during treatment with adalimumab in an open-label extension study (called the ADHERE trial—Additional Long-Term Dosing with HUMIRA to Evaluate Sustained Remission and Efficacy in Crohn’s disease).

## METHODS

### Study design

**Figure 1:**
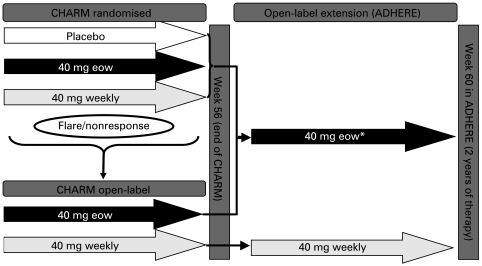
Study design. Flare was defined as a recurrence of very active disease (Crohn’s disease activity index (CDAI) increase ⩾70 points after week 4 and a CDAI >220). Non-response was defined as a failure to achieve 70-point response at any visit on or after week 12. *Option to adjust dosage to weekly for flare/non-response. ADHERE, Additional Long-Term Dosing with HUMIRA to Evaluate Sustained Remission and Efficacy in Crohn’s disease; CHARM, Crohn’s Trial of the Fully Human Antibody Adalimumab for Remission Maintenance; eow, every other week.

Detailed CHARM study methodology was reported previously.^6^ CHARM was a 56-week, multicentre, randomised, double-blind, placebo controlled trial with a 4-week open-label induction period. Patients successfully completing CHARM could enroll in an open-label extension study (ADHERE) (fig 1). At baseline, patients received open-label adalimumab 80 mg subcutaneously followed by 40 mg at week 2. At week 4, all patients still enrolled were stratified by whether or not they achieved a clinical response (defined as achieving a decrease in the Crohn’s disease activity index (CDAI) of ⩾70 points compared with baseline). Patients were then randomly assigned within each strata in a 1 : 1 : 1 ratio to one of three treatment groups: adalimumab 40 mg eow, adalimumab 40 mg weekly, or placebo.^6^

At or after week 12, a patient with a flare or non-response could be switched to open-label treatment with 40 mg of adalimumab eow, which could be increased to 40 mg weekly if needed. Continued non-response on open-label adalimumab 40 mg weekly resulted in withdrawal from the study. A flare was defined as a recurrence of very active disease (CDAI increase ⩾70 points from week 4 and a CDAI >220). Non-response was defined as failure to achieve 70-point response at any visit at or after week 12.

All patients who successfully completed CHARM could enter the extension study (ADHERE). Patients who completed CHARM on placebo therapy received open-label adalimumab eow. Patients who had switched to open-label adalimumab eow or weekly continued on those regimens, and patients could switch from eow to weekly dosing in the event of flares or non-response. Of note, patients could not decrease their dosing intervals back to eow once they began receiving weekly dosing in either study. In addition, patient visits in ADHERE (weeks 0, 2, 4, 8 and 12 and then every 12 weeks) were much less frequent than in CHARM (every 2 weeks to week 8, every 4 weeks to week 20, every 6 weeks to week 32 and then every 8 weeks to study end in the original CHARM controlled trial).

### Patients

Patients all had moderately to severely active CD, defined as a CDAI score between 220 and 450.^12^ Enrollment with a history of infliximab treatment was permissible only if infliximab had been discontinued at least 12 weeks before the screening visit and the patient had experienced an initial response to the agent (as judged by the investigator). Demographic and baseline disease severity data, concomitant medication use at baseline, previous history of TNF antagonist use and smoking history were collected for the entire population of patients in CHARM, but only patients with draining fistulas at screening and baseline are included in the subgroup analyses reported here. Baseline fistula data, that is the number of fistulas and their location, were noted and recorded.

### Efficacy assessments

The primary endpoint in CHARM was clinical remission and the study was statistically powered based on predicted remission rates. Fistula healing and the mean number of draining fistulas per day were predetermined secondary endpoints in CHARM; however, the study was not powered for secondary endpoints. Patients classified as having fistulas were only those with draining fistulas at both screening and at baseline of CHARM. Fistulas were assessed for spontaneous drainage or drainage with gentle compression at each study visit. Complete fistula healing was defined as the absence of drainage under both these circumstances.

In addition to these standard assessments of fistula closure at set time points, a new, prespecified statistical method was used to determine the number of draining fistulas per day. Fistula closure rates for patients with fistulas at baseline were also stratified by baseline immunosuppressant use (yes or no), baseline CD-related antibiotic use (yes or no) and previous history of use of any TNF antagonist (yes or no), as well as the reason for the discontinuation of the previous TNF antagonist (loss of response, adverse reaction, both).

Two post-hoc analyses were conducted to assess the sustained effect of adalimumab on fistula efficacy to 2 years of treatment (week 60 of ADHERE). The first analysis evaluated long-term fistula healing to 2 years of adalimumab therapy. The second analysis evaluated the maintenance of fistula healing in all patients with healed fistulas at the end of CHARM. In the primary fistula analysis specified in the statistical analysis plan, complete fistula healing (also referred to as complete fistula closure) was defined as the absence of draining fistulas for at least the last two post-baseline evaluations on or before the study visit in CHARM. Because of the reduced frequency of visits in ADHERE, fistula healing was defined as occurring at the time point of the visit, rather than for at least two consecutive visits, as was the case for the primary CHARM fistula analysis. For consistency, this definition of healing at a single time point for the 2-year analysis was used starting from the baseline of CHARM.

### Safety assessments

For evaluation of safety in the subset of patients with fistulas at baseline, serious adverse events and other adverse events of interest were collected throughout the study and its extension.

### Statistical analysis

Fistula results were evaluated for all randomly assigned patients with draining fistulas at both baseline and screening visits. The prespecified statistical analysis plan stipulated that fistula data from both adalimumab treatment groups (weekly and eow) be combined (owing to the anticipated small sample sizes of patients with fistulas) and compared with the placebo group. All analyses were based on two-sided tests with α  =  0.05. Continuous variables were compared using analyses of covariance adjusted for baseline values and discrete variables were compared using χ^2^ tests.

To calculate the draining fistulas per day, a new statistical approach was developed a priori to express the total fistula burden of each individual patient over the course of their blinded participation in CHARM. Starting at week 0 and ending at week 56 (or study discontinuation), the number of draining fistulas at each pair of consecutive evaluations was averaged and multiplied by the elapsed days between the evaluations to obtain the number of draining fistula days. The total number of draining fistula days was summed from week 0 to week 56 (or study discontinuation) and divided by the total days in the study to obtain the average number of draining fistulas per day. The difference between each blinded adalimumab group and the placebo group was assessed with least-squares means from the analysis of variance with treatment as the only factor. Of note, the number of study days excluded days after discontinuation of double-blind study drug. Similarly, draining fistulas observed after study discontinuation or after the discontinuation of double-blind treatment were excluded from the analysis. If a missing evaluation had non-missing double-blind evaluations before and after it, the average of the two non-missing evaluations was used to estimate the missing evaluation. This analysis was conducted for all randomly assigned patients and randomly assigned responders (defined as patients who achieved ⩾70 point decreases in CDAI scores at week 4 in CHARM).

The first analysis of long-term fistula healing to 2 years of adalimumab therapy included only those patients who were randomly assigned to adalimumab in CHARM and who entered ADHERE (analysis 1). Data were summarised at each visit using an observed-case analysis and non-responder imputation (NRI). In the second analysis of maintenance of fistula healing and response, all patients with healed fistulas at the end of CHARM (ie, both adalimumab and placebo-treated patients) were followed into ADHERE (analysis 2). In this analysis, data were summarised at weeks 24 and 60 of ADHERE (representing approximately 18 months and 2 years of therapy, respectively) using both observed and NRI analyses. With NRI analyses, the assumption is that patients with missing data or those who had left the trial were failures for the endpoint in question (ie, fistula healing).

## RESULTS

### Patients

**Figure 2:**
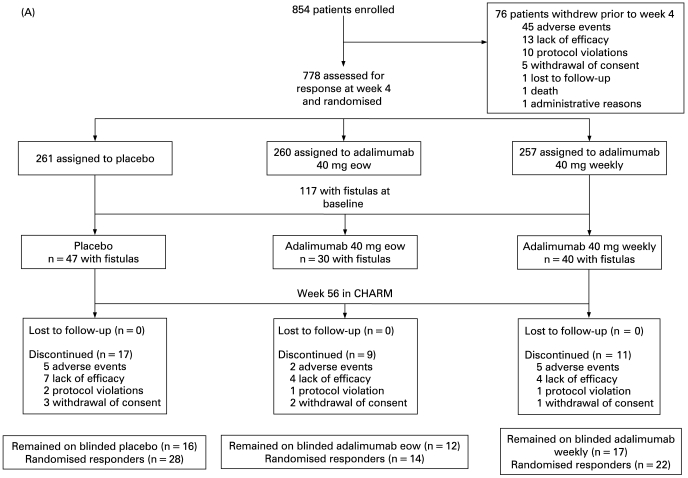

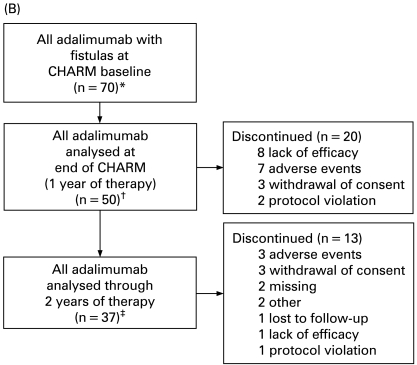

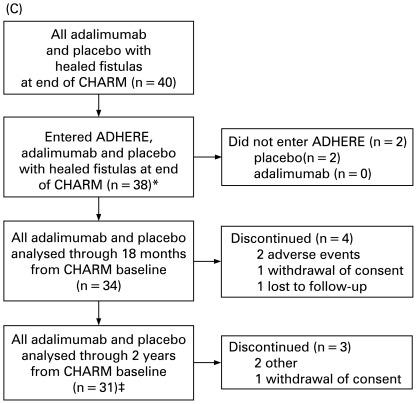
Patient disposition. (A) Patient disposition to week 56 of the Crohn’s Trial of the Fully Human Antibody Adalimumab for Remission Maintenance (CHARM). Randomly assigned responders achieved a 70-point or greater decrease in the Crohn’s disease activity index score at week 4 in CHARM. (B) Patient disposition for sustained fistula response over 2 years of adalimumab therapy in patients randomly assigned to adalimumab in CHARM and who enrolled in the Additional Long-Term Dosing with HUMIRA to Evaluate Sustained Remission and Efficacy in Crohn’s disease (ADHERE) study (analysis 1). (C) Patient disposition for sustained fistula response in all patients with healed fistulas at the end of CHARM (includes adalimumab and placebo-treated patients who entered ADHERE) (analysis 2). *Denominator for non-responder imputation (NRI) analyses. †Denominator for observed-case analysis at the end of CHARM. ‡Denominator for observed-case analysis to 2 years of therapy. eow, every other week.

The demographics and patient disposition for the overall CHARM patient population were previously described.^6^ Of the 778 patients randomly assigned at week 4 to receive placebo (n  =  261), adalimumab 40 mg eow (n  =  260) or adalimumab 40 mg weekly (n  =  257), there was a total of 117 patients with draining fistulas at screening and baseline (placebo, n  =  47; 40 mg eow, n  =  30; and 40 mg weekly, n  =  40; fig 2A). Figs 2B and C display the numbers of patients included in the long-term fistula efficacy analyses (to 2 years from CHARM baseline) and the reasons for exclusion from analysis. Baseline characteristics are presented in table 1.

**Table 1:** Baseline demographics and clinical characteristics

Characteristics	All patients (n = 854)	Patients with fistulas at baseline		
		Placebo (n = 47)	Adalimumab (n = 70)	All patients with fistulas (n = 117)
Male, no (%)	326 (38.2)	15 (31.9)	34 (48.6)	49 (41.9)
Age in years, mean (SD)	37.1 (11.9)	36.5 (10.1)	35.9 (12.2)	36.1 (11.4)
Baseline CDAI score, mean (SD)	313.1 (62.0)	308.0 (59.3)	318.4 (55.8)	314.3 (57.2)
CRP (mg/dl)				
Mean (SD)	2.3 (3.4)	3.6 (4.7)	2.8 (3.0)	3.1 (3.8)
Median (range)	0.9 (0.02–35.0)	2.3 (0.12–28.7)	1.8 (0.06–12.3)	1.9 (0.06–28.7)
CRP concentration ⩾1.0 mg/dl (10 mg/l), n (%)	407 (47.7)	32 (68.1)	42 (60.0)	74 (63.2)
Previous TNF antagonist exposure, n (%)	424 (49.6)	31 (66.0)	41 (58.6)	72 (61.5)
Concomitant medication, n (%)				
Any glucocorticoid*	376 (44.0)	20 (42.6)	29 (41.4)	49 (41.9)
Prednisone	244 (28.6)	14 (29.8)	18 (25.7)	32 (27.4)
Budesonide	100 (11.7)	9 (19.1)	2 (2.9)	11 (9.4)
Any immunosuppressive agent	399 (46.7)	26 (55.3)	31 (44.3)	57 (48.7)
Azathioprine	275 (32.2)	21 (44.7)	24 (34.3)	45 (38.5)
6-Mercaptopurine	81 (9.5)	4 (8.5)	3 (4.3)	7 (6.0)
Methotrexate	90 (10.5)	3 (6.4)	7 (10.0)	10 (8.5)
5-Aminosalicylates†	334 (39.1)	13 (27.7)	24 (34.3)	37 (31.6)
Current smoker, no (%)	303 (35.5)	18 (38.3)	20 (28.6)	38 (32.5)

*Includes betamethasone, budesonide, dexamethasone, deflazacort, cortisone, cloprednol, fluocortolone, glucocorticoids, hydrocortisone, methylprednisolone, prednisolone, prednisone, paramethasone and prednylidene. †Aminosalicylic acid, balsalazide, mesalazine, olsalazine, sulfasalazine. CDAI, Crohn’s disease activity index; CRP, C-reactive protein; TNF, tumour necrosis factor.

Baseline characteristics of the patients with draining fistulas at screening and baseline were similar compared with the remainder of the patients, with the exception of numerically greater C-reactive protein concentrations and a previous history of TNF antagonist exposure in the patients with fistulising disease. Baseline fistula data are presented in table 2.

**Table 2:** Baseline fistula data

Characteristic	Placebo (n = 47)	Adalimumab 40 mg eow (n = 30)	Adalimumab 40 mg weekly (n = 40)
Draining cutaneous fistulas,* no (%)			
One	30 (63.8)	19 (63.3)	23 (57.5)
Two	7 (14.9)	6 (20.0)	7 (17.5)
Three	6 (12.8)	1 (3.3)	5 (12.5)
Four	4 (8.5)	4 (13.3)	5 (12.5)
Perianal fistulas,† no (%)			
One	29 (64.4)	19 (63.3)	21 (55.3)
Two	7 (15.6)	6 (20.0)	7 (18.4)
Three	6 (13.3)	1 (3.3)	5 (13.2)
Four	3 (6.7)	4 (13.3)	5 (13.2)

*Draining cutaneous fistulas includes perianal fistulas (n  =  113) and abdominal fistulas (n  =  4). †n  =  45 for placebo; n  =  30 for 40 mg adalimumab eow; n  =  38 for 40 mg adalimumab weekly. eow, every other week.

### Efficacy

**Figure 3:**
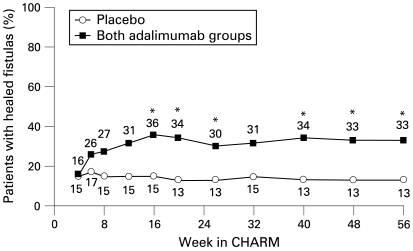
Percentage of patients with fistulas at baseline (adalimumab every other week (eow) and weekly combined, n  =  70; placebo, n  =  47) who had complete fistula healing over time in the Crohn’s Trial of the Fully Human Antibody Adalimumab for Remission Maintenance (CHARM; non-responder imputation analysis). *p<0.05 for combined adalimumab groups (40 eow and 40 mg weekly) compared with placebo for the intention-to-treat population. Fistula efficacy defined as no draining fistulas at the last two consecutive post-baseline evaluations in the double-blind period before and on that visit.

Adalimumab therapy was associated with progressive increases in fistula closure over time, with separation in the rates of complete closure between placebo and adalimumab groups evident as early as 2 weeks after randomisation. Statistically significant differences in fistula closure between placebo and adalimumab were first observed at 16 weeks (fig 3). Baseline immunosuppressant or CD-related antibiotic use had no apparent effect on the rates of fistula closure in patients receiving adalimumab or placebo at weeks 26 or 56, although the numbers of patients in each group were relatively small (table 3). In addition, whether patients were naive to or experienced with TNF antagonists before receiving adalimumab did not appear to effect fistula healing (data not shown).

**Table 3:** Percentage of patients with no draining fistulas at weeks 26 and 56 for all patients with fistulas at baseline (n  =  117) stratified by baseline concomitant therapies

Subgroup	No draining fistulas*	
	Week 26 % of patients	Week 56 % of patients
No baseline immunosuppressant use		
Placebo (n = 21)	19	19
Both adalimumab groups (n = 39)	33 (p = 0.369)	36 (p = 0.241)
Baseline immunosuppressant use		
Placebo (n = 26)	8	8
Both adalimumab groups (n = 31)	26 (p = 0.092)	29 (p = 0.051)
No baseline CD-related antibiotic use		
Placebo (n = 28)	14	14
Both adalimumab groups (n = 44)	32 (p = 0.162)	36 (p = 0.059)
Baseline CD-related antibiotic use		
Placebo (n = 19)	11	11
Both adalimumab groups (n = 26)	27 (p = 0.264)	27 (p = 0.264)

*Patients who had no draining fistulas at the last two post-baseline evaluations in the double-blind period on or before the week 26 or week 56 visits were classified as no; otherwise, patients were classified as yes. p Values from χ^2^ tests comparing both adalimumab groups combined versus placebo. CD, Crohn’s disease.

**Figure 5:**
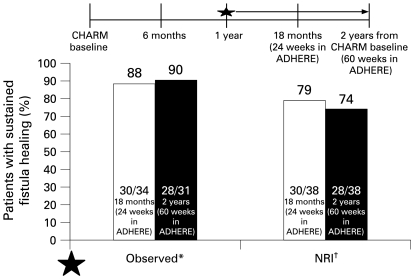
Long-term maintenance of fistula healing (absence of drainage from all fistulas, either spontaneously or upon gentle compression) in patients with healed fistulas at the end of the Crohn’s Trial of the fully Human Antibody Adalimumab for Remission Maintenance (CHARM; placebo, every other week (eow) and weekly; n  =  40).*Observed analysis: two placebo-treated patients with healed fistulas did not enter Additional Long-Term Dosing with HUMIRA to Evaluate Sustained Remission and Efficacy in Crohn’s disease (ADHERE) study; data on fistula outcomes were missing for four patients at 24 weeks in ADHERE and seven patients at 60 weeks in ADHERE. †Non-responder imputation analysis: two placebo-treated patients with healed fistulas did not enter ADHERE.

**Figure 4:**
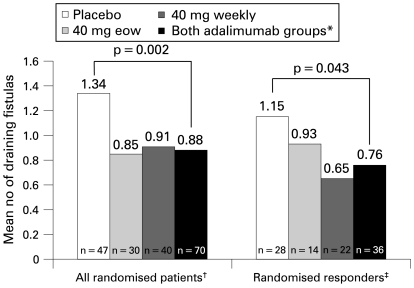
Mean number of draining fistulas per day during double-blind period. *Statistical analyses were not performed on individual adalimumab groups, per prespecified statistical plan. †Intention-to-treat population of patients with draining fistulas at screening and baseline visits. ‡Randomised responder population (⩾70 point decrease in Crohn’s disease activity index score at week 4 in the Crohn’s Trial of the Fully Human Antibody Adalimumab for Remission Maintenance) of patients with draining fistulas at screening and baseline visits. eow, every other week; n, number of patients with fistulas at baseline.

The new statistical methodology for determining fistula burden was used to calculate the number of draining fistulas per day during the double-blind period. For all randomly assigned patients and also all randomly assigned responder patients (those with week 4 CDAI decreases ⩾70 points vs baseline), there were significant decreases in the mean number of draining fistulas per day among adalimumab-treated patients compared with placebo-treated patients during the double-blind treatment period (fig 4). For all randomly assigned patients, the mean number of draining fistulas per day was 1.34 for placebo compared with a mean of 0.88 for the combined adalimumab groups (p = 0.002). Results were similar for the randomly assigned responders (mean 1.15 for placebo, mean 0.76 for adalimumab groups combined, p = 0.043). The effect of adalimumab on the number of draining fistulas in each subgroup (based on immunosuppressant, antibiotic, or previous TNF antagonist exposure) was similar to that observed for the overall placebo and adalimumab groups (data not shown).

Fistula healing over time for all patients with fistulas who were randomly assigned to adalimumab is provided in table 4 (observed analysis). Of the adalimumab-treated patients with fistulas at baseline (n  =  70) approximately 60% (22/37) had healed fistulas after 2 years of therapy (table 4). Long-term fistula healing over time using NRI analysis is provided in table 5. Because patients in CHARM and ADHERE were managed according to inflammatory activity and therapy dosage changes were not predicated on fistula response (fistula is part of CDAI, which is an inflammatory scale assessing disease activity), NRI is a very conservative approach to the data analysis.

**Table 4:** Long-term efficacy of fistula healing with adalimumab to 2 years from CHARM baseline: observed analysis*

Time point	Observed analysis n/No (%)
6 Months in CHARM	32/58 (55)
1 Year in CHARM	29/50 (58)
24 Weeks in ADHERE	25/42 (60)
36 Weeks in ADHERE	26/40 (65)
48 Weeks in ADHERE	23/37 (62)
60 Weeks in ADHERE†	22/37 (59)

*Includes all adalimumab-treated patients with fistulas at the baseline of the Crohn’s Trial of the Fully Human Antibody Adalimumab for Remission Maintenance (CHARM) remaining in CHARM or the Additional Long-Term Dosing with HUMIRA to Evaluate Sustained Remission and Efficacy in Crohn’s disease study (ADHERE; open-label extension) at the time points specified. Fistula efficacy defined as complete healing/closure of draining fistulas at time point since baseline of CHARM. †60 weeks in ADHERE represents approximately 2 years of adalimumab therapy.

**Table 5:** Long-term efficacy of fistula healing with adalimumab to 2 years from CHARM baseline: NRI analysis*

Time point	NRI (n = 70)
	No (%)
6 Months in CHARM	32 (46)
1 Year in CHARM	29 (41)
24 Weeks in ADHERE	25 (36)
36 Weeks in ADHERE	26 (37)
48 Weeks in ADHERE	23 (33)
60 Weeks in ADHERE†	22 (31)

*Includes all adalimumab-treated patients (every other week and weekly groups combined, N  =  70) with fistulas at baseline of the Crohn’s Trial of the Fully Human Antibody Adalimumab for Remission Maintenance (CHARM). Fistula efficacy defined as complete healing/closure of draining fistulas at time point since baseline of CHARM. †60 weeks in the Additional Long-Term Dosing with HUMIRA to Evaluate Sustained Remission and Efficacy in Crohn’s disease (ADHERE) study represents approximately 2 years of adalimumab therapy. NRI, non-responder imputation.

Long-term maintenance of fistula healing rates for patients with healed fistulas at the end of CHARM (n  =  40) are provided in fig 5. Of these patients, 90% (28/31) maintained healed fistulas after one additional year of treatment in ADHERE (2 years from CHARM baseline, observed analysis). In the NRI analysis, 75% of patients maintained healing to 2 years of therapy.

### Safety

Adalimumab was well tolerated for up to 56 weeks of treatment in patients with fistulas at baseline (table 6), similar to the findings for all randomly assigned patients reported by Colombel *et al*.^6^ Safety data in the fistula cohort to 2 years from CHARM baseline are presented in table 7. With respect to the serious events of abscess, eight events were reported in the 70 adalimumab-treated patients in the fistula cohort who received 2 years of therapy; one additional event was reported in a patient originally randomly assigned to placebo.

**Table 6:** Summary of safety in adalimumab and placebo-treated patients with fistulas to 56 weeks in CHARM

Event	Placebo (n = 47)	All adalimumab (n = 70)
	No (%)	No (%)
Adverse event	38 (80.9)	59 (84.3)
Serious adverse event	5 (10.6)	9 (12.9)
Adverse event leading to discontinuation of study medication	3 (6.4)	4 (5.7)
Infectious adverse event	16 (34.0)	31 (44.3)
Serious infectious adverse event*	2 (4.3)	5 (7.1)
Abscess (all)	5 (10.6)	8 (11.4)
Malignant neoplasm	0	0
Injection-site reaction (all)	2 (4.3)	3 (4.3)
Opportunistic infection†	1 (2.1)	0
Congestive heart failure	0	0
Demyelinating disorder	0	0
Death	0	0

*Both of the placebo-treated patients had an abdominal abscess. The serious infectious adverse events for the five adalimumab-treated patients were a pulmonary embolus with pneumonia (n  =  1); intra-abdominal abscess (n  =  1); perianal abscess (n  =  2) and scrotal abscess (n  =  1). †Oral candidiasis. CHARM, Crohn’s Trial of the fully Human Antibody Adalimumab for Remission Maintenance.

**Table 7:** Summary of safety in cohort of patients with fistulas to 2 years from CHARM baseline

Event	All patients with fistula (adalimumab and placebo) (n = 117)	All adalimumab patients with fistula (eow and weekly combined) (n = 70)
	No (%)	No (%)
Adverse event	112 (95.7)	70 (100)
Serious adverse event	33 (28.2)	22 (31.4)
Adverse event leading to discontinuation of study medication	19 (16.2)	13 (18.6)
Infectious adverse event	76 (65.0)	53 (75.7)
Serious infectious adverse event	13 (11.1)*	10 (14.3)†
Serious gastrointestinal adverse event	15 (12.8)‡	13 (18.6)§
Malignant neoplasm	1 (0.9)	0
Injection-site pain	7 (6.0)	4 (5.7)
Opportunistic infection	0	0
Congestive heart failure	0	0
Demyelinating disorder	0	0
Death	0	0

*Fifteen events were reported in 13 patients: nine events of abscess (four perianal, two abdominal, one rectal, one scrotal, one not specified), one event of clostridial infection, one event of sepsis, one event of pneumonia, one event of tuberculosis, one event of otitis media and one event of post-procedural infection. †Twelve events were reported in 10 patients: eight events of abscess (three perianal, two abdominal, one rectal, one scrotal, one not specified), one event of clostridial infection, one event of pneumonia, one event of tuberculosis and one event of otitis media. ‡Seventeen events were reported in 15 patients: 10 events of Crohn’s disease (CD), four events of obstruction (two intestinal, two small intestinal), two events of fistula (one anal, one intestinal) and one event of abdominal mass. §Fifteen events were reported in 13 patients: eight events of CD, four events of obstruction (two intestinal, two small intestinal), two events of fistula (one anal, one intestinal), and one event of abdominal mass. CHARM, Crohn’s Trial of the fully Human Antibody Adalimumab for Remission Maintenance.

## DISCUSSION

These data extend and define the beneficial effect of adalimumab on fistula healing in patients with CD and provide the first long-term fistula healing results with an anti-TNF therapy. This study also introduces and utilises a new methodology for evaluating the effect of treatment in patients with fistulising disease. The demographic characteristics of patients with fistulising disease did not differ substantially from the overall study population in CHARM. In this subgroup of patients whose CD was often difficult to manage because of fistulising disease, adalimumab still demonstrated a consistent and significant reduction of draining fistulas when compared with placebo in patients with moderate to severe CD. The effect of adalimumab on fistula closure was durable, as demonstrated by three-quarters of the adalimumab-treated patients (NRI; 90% with observed analyses) who had healed fistulas at the end of CHARM maintaining closure following an additional year of open-label therapy.

The efficacy of adalimumab was consistent in subgroups of patients with fistulas who were or were not receiving concomitant therapy with immunosuppressive agents or antibiotics, as well as in patients naive to and experienced with TNF antagonist therapy. However, caution should be exercised in interpreting these analyses as a result of the small numbers of patients in each subgroup.

**Figure 6:**
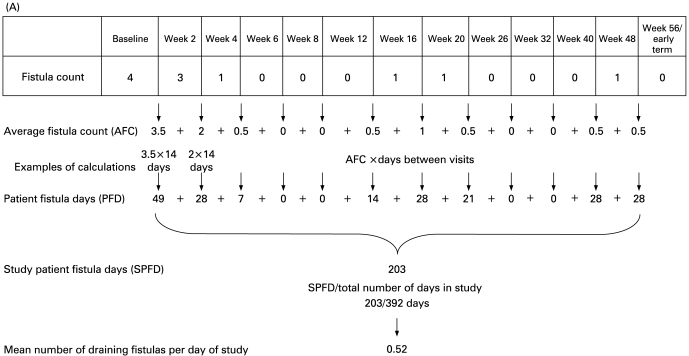

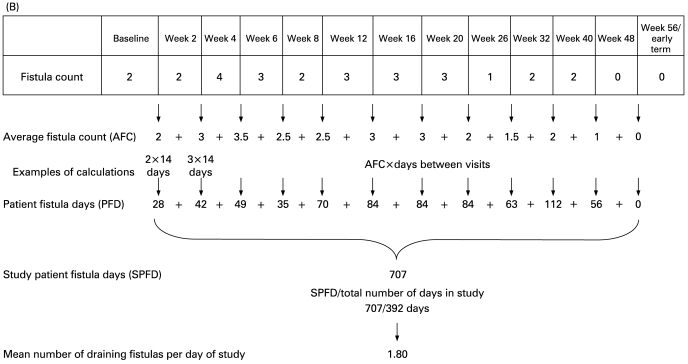
Sample calculation of the mean number of draining fistulas per day. (A) Hypothetical patient 1—minimal disease activity yet not achieving complete healing* at either week 26 or week 56. (B) Hypothetical patient 2—significant disease activity yet achievement of complete healing* at week 56. *Complete healing, no draining fistulas for at least the last two post-baseline evaluations in the double-blind period.

In addition to standard assessments of fistula closure at set time points, a new, prespecified statistical method was used to determine the number of draining fistulas per day. Specific examples of the statistical analysis are depicted in fig 6. With the definition of complete fistula healing used in this analysis, hypothetical patient 1 (fig 6A) would have been assessed as not having successful closure of fistulas at either individual time point (week 26 or week 56), whereas hypothetical patient 2 (fig 6B) would have been assessed as having successful closure at week 56. With the new method of measuring fistulas per day reported here, it becomes evident that hypothetical patient 1 actually had a lower fistula burden over the study period than did hypothetical patient 2. Evaluation of “fistulas per day” more accurately reflects disease activity over the entire study period, instead of focusing on fistula counts at single points in time as does the definition of fistula healing. By providing a longitudinal perspective, this new methodology may yield a more accurate picture of the total fistula experience of each individual patient over the full course of participation in the study. This analysis, which facilitates the comparison of subgroups of patients within an individual study, was prespecified in the statistical analysis plan for the CHARM study. One limitation of this method is that between the visits, the numbers of draining fistulas on each day are assumed to remain the same and approximate the average number of draining fistulas observed at each visit. This assumption requires validation in appropriately constructed clinical trials.

Infliximab is the only other TNF antagonist shown to be effective in inducing and maintaining fistula closure.^4^ ^5^ In the ACCENT II study (Crohn’s Disease Clinical Study Evaluating Infliximab in a New Long-Term Treatment Regimen II), 36% of patients who had responded to induction therapy and received maintenance infliximab therapy maintained complete fistula closure compared with 19% of patients receiving placebo (p = 0.009).^5^ In ACCENT II, patients were included based on a minimal duration of fistula activity; only patients who responded to initial induction therapy were included in the analysis of maintenance efficacy.^5^ In CHARM, patients were included based on the overall activity of their CD and not on fistula activity, and all patients with fistulas were included in the analysis irrespective of initial response to adalimumab. Despite these differences, the fistula closure rates from ACCENT II at week 54 are generally comparable to the fistula closure rates observed with adalimumab treatment at week 56. In two studies of certolizumab pegol (PRECiSE 1 and 2), a pegylated Fab′ fragment of an anti-TNF monoclonal antibody, the effect of certolizumab on fistula closure was not statistically significantly different compared with placebo.^13^ ^14^

Patients with fistulas at baseline tolerated sustained adalimumab treatment well. The safety profile of adalimumab in CHARM was consistent with studies of adalimumab in rheumatological disorders and with previous studies of patients with CD. The safety of TNF antagonists in patients with fistulising disease is of concern, because of the greater risk of infection at baseline (especially abscesses) for these patients.^15^ In the CHARM fistula cohort, there were no differences in the rates of adverse events, including infectious adverse events and, specifically, abscesses, in patients who received placebo compared with those who received adalimumab at either dosage. This observation is consistent with previous reports.^16^

## CONCLUSIONS

Adalimumab therapy resulted in a significant decrease in the number of draining fistulas per day compared with placebo. Significant and complete healing of draining fistulas was observed in adalimumab-treated patients, and long-term healing of draining fistulas was maintained over 2 years from the baseline of the CHARM study. Adalimumab was well tolerated, with a safety profile consistent with previous studies in patients with CD.
